# Reduction of Allergic Lung Disease by Mucosal Application of *Toxoplasma gondii*-Derived Molecules: Possible Role of Carbohydrates

**DOI:** 10.3389/fimmu.2020.612766

**Published:** 2021-03-10

**Authors:** Elke Korb, Mirjana Drinić, Angelika Wagner, Nora Geissler, Aleksandra Inic-Kanada, Roman Peschke, Anja Joachim, Ursula Wiedermann, Irma Schabussova

**Affiliations:** ^1^ Institute of Specific Prophylaxis and Tropical Medicine, Medical University of Vienna, Vienna, Austria; ^2^ Department of Pathobiology, Institute of Parasitology, University of Veterinary Medicine Vienna, Vienna, Austria

**Keywords:** *Toxoplasma gondii*, tachyzoites lysate antigen, allergic airway inflammation, immunomodulation, deglycosylation, hygiene hypothesis, parasites, carbohydrates

## Abstract

**Background:**

The hygiene hypothesis suggests a link between parasitic infections and immune disorders, such as allergic diseases. We previously showed that infection with *Toxoplasma gondii* or systemic application of *T. gondii* tachyzoites lysate antigen (TLA) in a prophylactic, but not therapeutic protocol, prevented allergic airway inflammation in mice. Here we tested the effect of prophylactic and therapeutic application of TLA *via* the mucosal route.

**Methods:**

Mice were intranasally treated with TLA either i) prior to sensitization, ii) during sensitization and challenge, or iii) after sensitization with ovalbumin (OVA). Recruitment of inflammatory cells to the lung, cytokine levels in restimulated lung and spleen cell cultures as well as levels of OVA-specific antibodies in serum were measured. In parallel, the effect of native TLA, heat-inactivated (hiTLA) or deglycosylated TLA (dgTLA) on sensitized splenocytes was evaluated *ex vivo*.

**Results:**

When applied together with OVA i) during systemic sensitization and local challenge or ii) exclusively during local challenge, TLA reduced infiltration of eosinophils into the lung, OVA-specific type 2 cytokines in restimulated lung cell cultures, and partially, type 2 cytokines in restimulated spleen cell cultures in comparison to allergic controls. No beneficial effect was observed when TLA was applied prior to the start of sensitization. Analysis of epitope sugars on TLA indicated a high abundance of mannose, fucose, N-acetylglucosamine, and N-acetylgalactosamine. Deglycosylation of TLA, but not heat-inactivation, abolished the potential of TLA to reduce type 2 responses *ex vivo*, suggesting a significant role of carbohydrates in immunomodulation.

**Conclusion:**

We showed that mucosal application of TLA reduced the development of experimental allergy in mice. The beneficial effects depended on the timing of the application in relation to the time point of sensitization. Not only co-application, but also therapy in sensitized/allergic animals with native TLA reduced local allergic responses. Furthermore, we show that TLA is highly glycosylated and glycoconjugates seem to play a role in anti-allergic effects. In summary, given the powerful modulatory effect that TLA exhibits, understanding its exact mechanisms of action may lead to the development of novel immunomodulators in clinical application.

## Introduction

Allergic diseases of the airways affect millions of people and the prevalence has been increasing continuously. Allergic asthma is a heterogeneous and complex inflammatory disease of the airways that develops upon allergic sensitization with allergens, such as house dust mites, cockroaches, animal dander, grass and tree pollens, and fungal spores (1). These inhaled allergens stimulate the production of type 2 cytokines, such as IL-4, IL-5, and IL-13, leading to airway eosinophilia, airway hyperresponsiveness, mucus hypersecretion, and elevated IgE in serum (1, 2).

The lack of preventative, curative, and disease modifying strategies for asthma establishes substantial unmet medical need. Although symptoms can be treated e.g. with inhaled glucocorticosteroids, bronchodilators, or monoclonal anti-IgE antibodies (3–5), specific immunotherapy (SIT) is the only available curative treatment of allergy, inducing desensitization and long-term allergen-specific immunological tolerance (6). In patients with mild to moderate asthma, SIT can reduce allergic symptoms, such as dyspnea, cough, wheeze, chest tightness, and medication requirements, but studies showing the effects on overall lung function have been inconclusive (6–9). A noteworthy drawback of current SIT is the lack of high-quality allergen extracts with well-defined composition (10). Another clear disadvantage is that SIT may lead to novel sensitization to antigens in the formulation, or even to anaphylactic reactions (11).

*Toxoplasma gondii* is a ubiquitous, obligate intracellular protozoan parasite which replicates sexually in felids (12, 13). Shed oocysts can be transmitted *via* the fecal-oral route, while tissue cysts containing bradyzoites can be ingested upon the consumption of undercooked meat, infecting warm-blooded animals including humans (13, 14). Upon ingestion, oocysts release sporozoites to the lumen of the gut, which in turn differentiate into tachyzoites (15). Infection with *T. gondii* remains mostly asymptomatic in healthy individuals. However, severe disease has been reported in immunocompromised patients and in congenitally infected newborns (13, 14).

Since the postulation of the hygiene hypothesis in the late 1980s (16), multiple studies suggested a link between the prevalence of allergic diseases and increased hygiene standards, alongside an early use of antibiotics, reduced bacterial exposure, and low incidences of parasitic infections in industrialized countries (17–19). Epidemiological studies reported that humans that were infected with *T. gondii* exhibited reduced prevalence of allergic diseases (20). Indeed, we and others confirmed these epidemiological findings by showing that infections with *T. gondii* reduced allergic airway inflammation in mice (21, 22). Of note, the prevention of allergy was achieved also by non-infectious molecules derived from *T. gondii*, such as oocyst lysate antigen (OLA), when applied intraperitoneally and in the presence of Freund’s complete adjuvant GERBU (23).

Harvesting *T. gondii* oocysts for the preparation of OLA requires passaging through feline hosts, such as cats. The yields are relatively low in proportion to the efforts and collected material may be contaminated with bacterial or host-derived components (24). On the other hand, culturing *T. gondii* tachyzoites *in vitro* in Vero cells is ethically, and also economically, advantageous compared to harvesting of oocysts. Furthermore, *in vitro* culturing enables high yields of tachyzoites while posing a significantly reduced risk of contamination (25, 26). Recently, we have shown that not only OLA, but also adjuvanted tachyzoite lysate antigen (TLA), applied *via* the parenteral route, reduced experimental allergic inflammation in mice (24).

The mucosal application of immunomodulatory substances or drugs, e.g. *via* the intranasal route, offers several benefits over parenteral application, such as a facilitated, needle-free administration, thereby reducing the need for trained medical personnel, as well as a lung-targeted delivery (27, 28). Along these lines, we previously demonstrated the high potential of the intranasal route to reduce allergic responses in the lung by treating mice with probiotic bacterial strains (29–31).

To date, there is only limited understanding which components of TLA are essential players in allergy-prevention. TLA consists mostly of proteins, carbohydrates (32, 33), and membrane-associated lipids (34, 35). We previously showed that compared to native and heat-inactivated TLA, sodium metaperiodate-treated deglycosylated TLA exhibited reduced potential to induce cytokines *in vitro* in naïve splenocytes (24).

Here we tested the effect of intranasally applied TLA on experimental allergic airway inflammation. Furthermore, we investigated whether mucosal immunological tolerance could be established in sensitized mice in a therapeutic treatment protocol. Finally, we characterized the glycosylation pattern of TLA and evaluated if carbohydrates play an important role in TLA-induced immunomodulation.

## Material and Methods

### Preparation of TLA

*T. gondii* tachyzoites (strain S-48) were cultivated in Vero cells, harvested and TLA extracts were prepared as described previously (24). Briefly, TLA was prepared by three freeze-thaw cycles in liquid nitrogen, followed by sonication and subsequent centrifugation at 10,000 x *g* (60 min at 4°C). The supernatant was sterile filtered (0.22 µm Millex^®^GV Filter Unit; Merck Milipore) and its protein concentration was assessed with a BCA Protein Assay Reagent kit (Pierce Peribo). Endotoxin levels were determined by Limulus Amoebocyte Lysate (Endpoint Chromogenic LAL Assay; Lonza LTD) and were below 0.1 EU in 1 µg of extract. TLA was stored at −80°C until further use.

### Heat-Inactivation and Sodium Metaperiodate Treatment

TLA was heat-inactivated by incubation for 15 min at 95°C (hiTLA) and deglycosylation (dgTLA) was performed by sodium metaperiodate treatment according to a modified protocol (36). Briefly, 3 mg TLA (1 mg/ml in PBS) were mixed with 3 ml 100 mM sodium metaperiodate in 100 mM acetate buffer, at pH = 4.5. TLA was incubated at 37°C for 30 min in the dark. The oxidation reaction was stopped with 300 ml 0.5 x PBS. The volume was then reduced to 5 ml by centrifugation at 2,900 x *g* at 4°C using Ultracel^®^-3K centrifugal filters (Amicon^®^ Ultra-15; Merck Millipore). Volume was further decreased with a Vacuum Concentrator Centrifuge (Univapo 150H; Uniequip). The protein concentration was assessed as above and modification of glycan moieties was verified with a Western blot using biotinylated Concanavalin A (ConA) as described below.

### SDS-Page and Western Blot

10 µg TLA and dgTLA were separated with SDS-PAGE according to the manufacturer’s protocol using NuPAGE™ 4–12% Bis-Tris Protein Gels (10-well and 12-well; Invitrogen) and PagerRuler™ Prestained Protein Ladder (10–180 kDa; Thermo Scientific). Protein bands were transferred to nitrocellulose membranes (Whatman Protram Nitrocellulose Transfer Membrane; Whatman) and a Western blot was performed using biotinylated *Anguilla anguilla* agglutinin (AAA) (1 mg/ml, 1:5,000 dilution), biotinylated *Lycopersicon esculentum* lectin (LEL) (1 mg/ml, 1:5,000 dilution), biotinylated *Wisteria floribunda* agglutinin (WFA) (0.5 mg/ml, 1:2,500 dilution), biotinylated ConA (1 mg/ml, 1:5,000 dilution), or biotinylated *Ulex europaeus* agglutinin I (UEA-I) (1 mg/ml, 1:5,000 dilution). The membranes were subsequently incubated with monoclonal anti-biotin–alkaline phosphatase antibody produced in mouse (1:10,000 dilution; Sigma) and stained with SIGMAFAST™ BCIP^®^/NBT staining tablets (Sigma).

### Enzyme Linked Lectin Assay

Abundance of epitope sugars was determined by a modified enzyme-linked lectin assay (ELLA) protocol as described elsewhere (37). Briefly, plates were coated with 5 µg TLA in 200 µl PBS overnight at 4°C, blocked for 2 h at room temperature using 3% BSA in TBS-buffer (20 mM Tris, 150 mM NaCl, 1 mM CaCl_2_, and 1 mM MgCl_2_, pH = 7.6), and subsequently incubated for 1 h with 1 µg biotinylated lectins in 100 µl TBS and 0.05% Tween-20 (TBS-T). The following lectins were used: *Griffonia simplicifolia* (GS II), Wheat germ agglutinin (WGA), WFA, *Maackia amurensis* agglutinin (MAA), *Galanthus nivalis* agglutinin (GNA), *Dolichos biflorus* agglutinin (DBA), *Bauhinia purpurea* agglutinin (BPA), LEL, *Ricinus communis* agglutinin II (RCA II), UEA-I, *G. simplifolica* 1B4 (GS1B4), phytohemagglutinin (PHA-L), AAA, *Helix pomatia* agglutinin (HPA), Peanut agglutinin (PNA), ConA and *Sambucus nigra* agglutinin (SNA). Next, plates were incubated with horseradish peroxidase avidin (HRP-avidin) (1:250, eBioscience™ Avidin HRP; Invitrogen™; ThermoFisher Scientific) in 50 µl TBS-T and epitope sugars were detected with brief incubation with 3,3′,5,5′-tetramethylbenzidine (TMB). The colorimetric reaction was stopped with 0.18 M H_2_SO_4_ and absorption was measured at 450 nm with a SparkControl Magellan plate reader (Tecan).

### Mice

Female, 6 to 8 weeks old wild-type BALB/c mice were purchased from Charles River (Sulzfeld, Germany). Mice were kept under conventional housing conditions. Experiments were approved by the Ethics Committee of the Medical University of Vienna and the Austrian Federal Ministry of Education, Science and Research (BMWFW-66.009/0358-WF/V/3b/2015 and BMBWF-66.009/0277-V/3b/2019).

### Experimental Design

On days 0 and 14 mice were intraperitoneally immunized with 10 µg ovalbumin (OVA; grade V; Sigma-Aldrich) in PBS and 67% (v/v) alum (Alu-Gel-S Suspension; Serva Electrophoresis) in a total volume of 150 µl or with PBS and alum alone. Mice were challenged intranasally with 100 µg OVA in PBS in a total volume of 30 µl or 30 µl PBS alone on days 21 to 23. For the prophylactic approach, mice were treated intranasally with 30 µg TLA in 30 µl PBS or PBS alone on days -7 to -5. For the co-application strategy, mice were treated intranasally with 30 µg TLA in 30 µl PBS or 30 µl PBS alone 30 min prior sensitization and challenge on days 0, 14, 21, 22, and 23. For the therapeutic setup, mice were administered intranasally 30 µg TLA or hiTLA in 30 µl PBS or PBS alone on days 18 to 20 and 30 min prior challenge on days 21 to 23. Before each challenge and TLA treatment, mice were anesthetized with 5% (v/v) isoflurane (Isocare; Inhalation vapor, Animalcare Ltd.) at an airflow rate of 3 L/min in a UniVet Porta anesthesia machine (Groppler Medizintechnik). In the co-application model, 24 h after the last challenge, airway hyperresponsiveness (AHR) was assessed with whole-body plethysmography (Buxco Electronics Inc., DSI). Mice were conscious and unrestrained while they were exposed to 0, 12.5, and 50 mg/mL of aerosolized methacholine (acetyl-β-methyl-choline chloride; Sigma-Aldrich) in PBS. AHR was expressed by the dimensionless parameter enhanced pause (PenH) as previously described (38).

### Differential Cell Counts in Bronchoalveolar Lavage Fluid (BALF)

Lungs were lavaged with 1 ml ice-cold PBS. BALF was centrifuged (300 × *g* for 5 min at 4°C). Pelleted cells were resuspended in PBS and 4 x 10^4 cells were spun onto microscope slides (800 x *g* for 3 min; Shandon Cytospin, Shandon Southern Instruments), air-dried and stained with hematoxylin and eosin (H&E; Hemacolor^®^, Merck). At least 130 cells (macrophages, eosinophils, lymphocytes, and neutrophils) per slide were counted under a light microscope (1000 x magnification; Nikon Eclipse, Nikon).

### Lung Cells Isolation and Stimulation *Ex Vivo*

Lungs of terminally anesthetized mice were excised and processed as described elsewhere (39). Briefly, lungs were minced and digested in 6 ml RPMI-1640 media (Gibco^®^, Thermo Fisher Scientific) containing 0.05 mg/ml Liberase TL (Roche) and 0.5 mg/ml DNAse (Sigma-Aldrich) for 45 min at 37°C in 5% CO_2_ atmosphere. Next, the digested tissue was forced through a 70 µm cell strainer and erythrocytes were lysed in 3 ml ACK Lysing Buffer (BioWhittaker^®^, Lonza) for 90 s. Lung cells were resuspended (5 x 10^6 cells/ml) in complete RPMI (RPMI-1640 containing 10% FCS, 2 mM mercaptoethanol, 2 mM L-glutamine and 100 µg/ml gentamycin; Sigma-Aldrich). One hundred microliter cell suspensions were plated into 96-well plates and incubated either with complete RPMI or with 100 µg/ml endotoxin-free OVA (Endo-Grade; Hyglos) in complete RPMI for 72 h at 37°C in 5% CO_2_ atmosphere. After incubation, supernatants were collected and analyzed for the production of cytokines (IL-4, IL-5, IL-13, and IFN-γ) with ELISA kits following the manufacturer’s instructions (Ready-SET-Go!™ Kit, eBioScience™, Thermo Fisher Scientific).

### Spleen Cells Isolation and Stimulation *Ex Vivo*

Spleens of terminally anesthetized mice were processed to single-cell suspensions. Briefly, spleens in 10 ml RPMI-1640 media were pressed through a metal net and the disrupted tissue was subsequently forced through a 70 µm cell strainer. Erythrocytes were lysed in 3 ml ACK Lysing Buffer for 60 s. Similar to lung cells, spleen cells were resuspended (5 x 10^6 cells/ml) in complete RPMI and restimulated and assessed for cytokine production following the same protocol that was used for lung cells, except for *ex vivo* restimulations analyzing the immunomodulatory properties of differently treated TLA. Here, 100 µl spleen cell suspensions of allergic mice were incubated with 50 µl complete RPMI or 0.75 µg native, heat-inactivated or deglycosylated TLA in 50 µl complete RPMI for 1 h at 37°C in 5 % CO_2_ atmosphere. Next, cells were incubated on top with either 50 µl complete RPMI or with 20 µg endotoxin-free OVA (Endo-Grade; Hyglos) in 50 µl complete RPMI for 72 h at 37°C in 5 % CO_2_ atmosphere. After incubation, supernatants were collected and analyzed for the production of cytokines (IL-4, IL-5, IL-10, and IFN-γ as above).

### Lung Histology

Lungs were infiltrated with 7.5% (v/v) formaldehyde for histology. Formalin-fixed lungs were dehydrated with a series of ethanol solutions, followed by xylene, and subsequently embedded in paraffin. Sections (3 µm) were stained either with H&E or with Periodic acid-Schiff (PAS; Sigma-Aldrich). The histological pathology score was evaluated according to Zaiss et al. (40) with modifications. Stained sections were scored according to following criteria regarding i) perivascular and peribronchiolar inflammation (H&E) (0 = no inflammation; 1 = single scattered leukocytes; 2 = aggregates less than 10 cells thick; 3 = aggregates more than 10 cells thick; 4 = numerous coalescing aggregates more than 10 cells thick) and ii) number of leukocytes in alveolar spaces (H&E) {0 = not present; 1 = rare [2 to 4 cells in 400 × HPF (high power field)]; 2 = moderate (5 to 10 cells); 3 = high (more than 10 cells)}. The numbers of PAS-positive mucus producing goblet cells in the bronchial epithelium were counted and expressed per one millimeter of basement membrane according to Skevaki et al. (41).

### Collection of Blood Serum

At the beginning of the experiment (day -7 or day 0) as well as on days 13 and 25, approximately 100 µl blood were collected by puncturing the facial vein. Serum was obtained by centrifuging the blood in microtainer^®^ SST™ tubes (BD) at 15,000 x *g* for 5 min. Serum was stored at −20°C until analysis.

### Detection of OVA-Specific Antibodies

Microtiter plates were coated with OVA (5 µg/ml; grade V) and blocked with 1 % (w/v) BSA and 0.05% (v/v) Tween in PBS for 6 h and subsequently incubated with BALF or with serum samples at 4°C overnight. BALF supernatant was tested neat and sera were diluted 1:2,000 for IgG1, 1:500 for IgG2a and 1:20 for IgE. The next day, plates were washed and incubated with rat-anti mouse IgG1, IgG2a or IgE (1:500, BD Pharmingen™) at 4°C overnight. On the next day, plates were washed and incubated with horseradish peroxidase-conjugated mouse anti-rat IgG (1:2,000; Jackson ImmunoResearch Laboratories Inc.) for 1 h at 37°C, followed by incubation for 1 h at 4°C. Plates were washed again and 1 mM ABTS (Sigma-Aldrich) in 70 mM citrate-phosphate buffer (pH = 4.2; Sigma-Aldrich) was added for colorimetric measurement. Absorption was measured at 405 nm with a SparkControl Magellan plate reader.

### Rat Basophil Leukemia (RBL) Cell-Based Assay

RBL cell mediator release assay was performed as described elsewhere (41). Briefly, RBL 2H-3 cells were plated into 96-well plates (4 x 10^4 cells/well) and incubated with serum samples (1:300) from the beginning and the end of the experiment for 2 h at 37°C in a 5 % CO_2_ atmosphere. Cells were washed with Tyrode’s buffer [137 mM NaCl, 5.6 mM D glucose, 2.7 mM KCl, 1.8 mM CaCl_2_, 1.1 mM MgCl_2_, 0.4 mM NaH_2_PO_4_, 12 mM NaHCO_3_, 10 mM HEPES and 0.1 % (w/v) BSA; pH = 7.4; Sigma-Aldrich] and degranulation of cells was induced by incubation with 0.3 µg/ml OVA in Tyrode’s buffer. Supernatants were analyzed for β-hexosaminidase content by incubation with 80 μM 4-methylumbelliferyl-N-acetyl-β-d-glucosaminide (Sigma-Aldrich) and measuring fluorescence at λex: 360 nm/λem: 465 nm with a SparkControl Magellan plate reader. Results show percentage of total β-hexosaminidase release after adding 1 % (v/v) Triton X-100 (Sigma-Aldrich) in ddH_2_O.

### Statistics

The comparison of cytokine levels and histopathology scoring of all treatment groups was performed with two-way analysis of variance, followed by Bonferroni’s multiple comparison test for the prophylactic model or followed by Tukey’s multiple comparison test for the co-application and therapy models. Significance between all treatment groups in BALF as well as multiple time points in RBL assays and serum antibody ELISA was assessed with two-way analysis of variance, followed by Bonferroni’s multiple comparison test. The comparison of all treatment groups of *ex vivo* TLA stimulation and the comparison of OVA-specific IgG2a in BALF was performed with one-way analysis of variance, followed by Tukey’s multiple comparison test. Statistical comparisons were performed using GraphPad Prism Software 7 (GraphPad Software Inc.). All data are shown as mean ± SEM. Significant differences were considered at P < 0.05 (*), P < 0.01 (**), P < 0.001 (***) and P < 0.0001 (****).

## Results

### Prophylactic Intranasal TLA Treatment of Mice Before OVA Sensitization and Challenge Does Not Reduce Allergic Airway Inflammation

**Figure 1:**
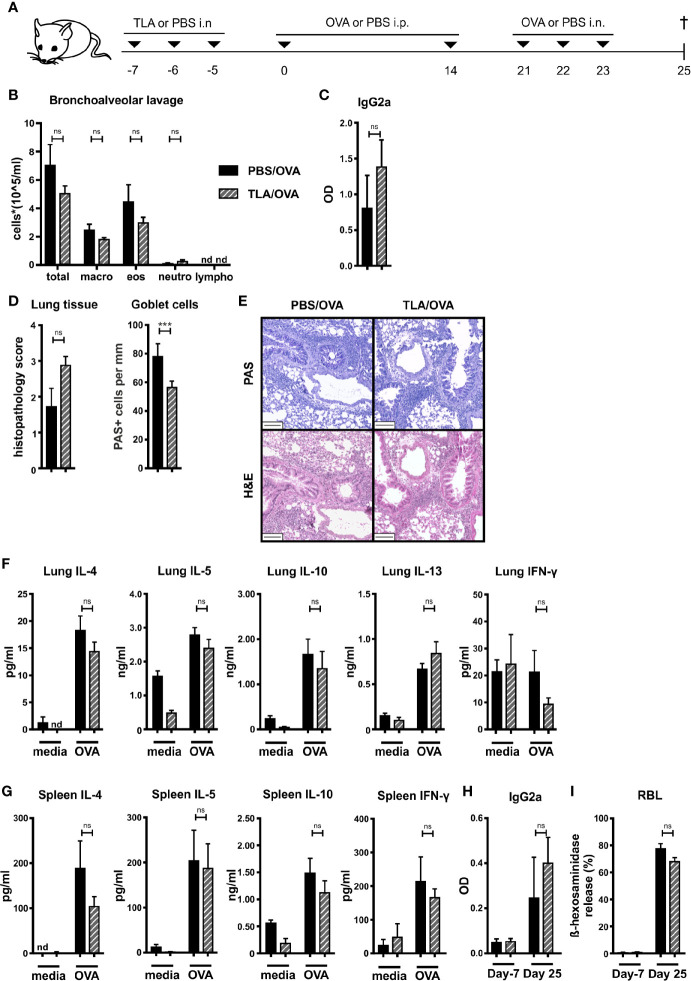
Prophylactic treatment with TLA fails to prevent allergic airway inflammation. **(A)** Experimental design. **(B)** Differential cell counts in bronchoalveolar lavage (BALF). **(C)** Levels of OVA-specific antibody IgG2a in BALF collected at the end of the experiment. **(D)** Average histopathology score and number of Periodic acid-Schiff (PAS)-positive goblet cells of lung sections. **(E)** PAS or hematoxylin and eosin (H&E)-stained lung sections from 1 representative example from each group (n = 5); scale bars, 100 µm. **(F)** Levels of IL-4, IL-5, IL-10, IL-13, and IFN-γ after medium and ovalbumin (OVA) restimulation of lung cells. **(G)** Levels of IL-4, IL-5, IL-10, and IFN-γ after medium and OVA restimulation of spleen cells. **(H)** Levels of OVA-specific antibody IgG2a in serum collected at the beginning and at the end of the experiment. **(I)** Release of β-hexosaminidase by rat basophil leukemia (RBL) cells. Graphs show results from 1 representative experiment from 2 independent experiments with 5 mice per group (**Figures 1B–I**). Error bars show mean ± SEM. *TLA,* tachyzoites lysate antigen; *OVA,* ovalbumin; *i.n.,* intranasal; *i.p.,* intraperitoneal; *macro,* macrophages; *eos,* eosinophils; *neutro,* neutrophils; *lympho*, lymphocytes; OD, optical density; *nd*, not detectable; *ns*, not significant; ***P < 0.001.

We tested the ability of TLA to reduce the development of experimental allergy when applied prophylactically *via* the mucosal route. Mice were sensitized by two i.p. injections of OVA in alum followed by three intranasal challenges of OVA one week later on days 21 to 23 (**Figure 1A**). In the OVA-sensitized and challenged control mice (PBS/OVA), high levels of macrophages and eosinophils and low levels of neutrophils and lymphocytes were detected in BALF (**Figure 1B**, **Supplementary Figure S1A**). Additionally, high levels of OVA-specific IgG2a in BALF (**Figure 1C**) and high numbers of inflammatory cells and PAS-positive goblet cells were detected in the lung of these mice (**Figures 1D, E** **)**. In parallel, restimulation of lung and spleen cell cultures with OVA led to production of high levels of type 2 cytokines IL-4, IL-5, and IL-13, and of IL-10 in comparison to cultures which were stimulated with media only (**Figures 1F, G** **)**. In lung cells, the levels of IFN-γ were similar upon restimulation with media or OVA (**Figure 1F**), while in spleens the levels of IFN-γ were increased upon restimulation with OVA compared to restimulation with media in the PBS/OVA group (**Figure 1G**). The level of sensitization in PBS/OVA mice was documented by increased OVA-specific IgG2a (**Figure 1H**) and OVA-specific β-hexosaminidase release after incubation of RBL cells with serum collected on day 25 compared with serum from day -7 (**Figure 1I**). Intranasal application of TLA on days -7 to -5 (**Figure 1A**) prior to allergic sensitization with OVA had no significant effect on the total counts of macrophages, eosinophils, neutrophils, and lymphocytes in the lung (**Figure 1B** **,** **Supplementary Figure S1A**). Similarly, the levels of OVA-specific IgG2a in BALF (**Figure 1C**), the histopathology score in the lung (**Figures 1D, E**), the production of OVA-induced cytokines in restimulated lung (**Figure 1F**) or spleen cell cultures (**Figure 1G**) were unaffected by TLA treatment. In serum, no significant changes of OVA-specific IgG2a levels (**Figure 1H**) were detected. Similarly, no effect of TLA-treatment on OVA-specific IgE-mediated β-hexosaminidase release by RBL cells was observed (**Figure 1I**). Of note, reduced numbers of PAS-positive goblet cells were detected in the lung in TLA-treated mice compared to allergic controls (**Figure 1D**).

### Intranasal Application of TLA Concurrent With OVA Sensitization and Challenge Reduces Allergic Airway Inflammation

**Figure 2:**
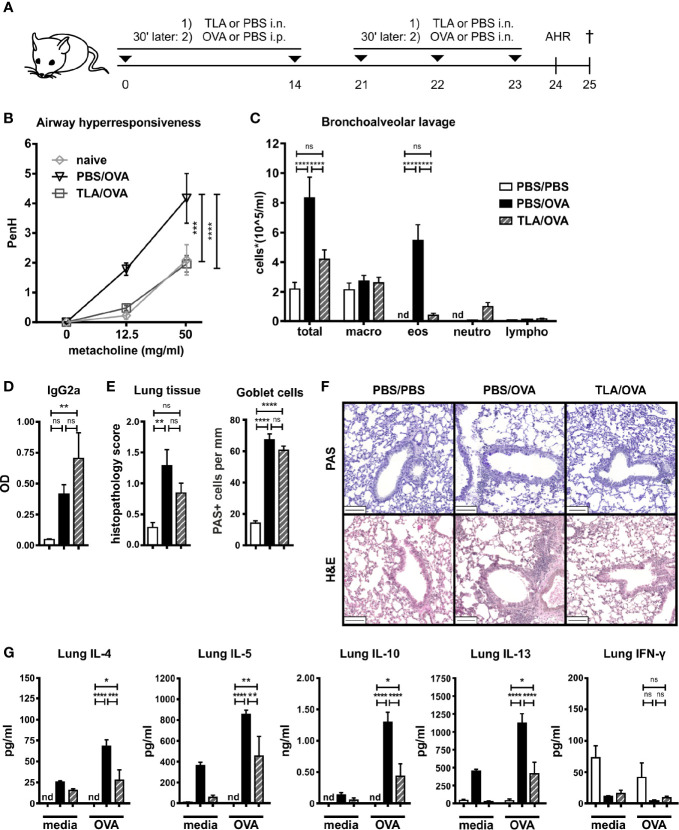
TLA reduces allergic airway inflammation in a co-application model. **(A)**, Experimental design. **(B)** Airway hyperresponsiveness in response to methacholine. **(C)** Differential cell count in bronchoalveolar lavage (BALF). **(D)** Levels of OVA-specific antibody IgG2a in BALF collected at the end of the experiment. **(E)** Average histopathology score and number of Periodic acid-Schiff (PAS)-positive goblet cells of lung sections. **(F)** PAS or hematoxylin and eosin (H&E)-stained lung sections from 1 representative example from each group (n = 5); scale bars, 100 µm. **(G)** Levels of IL-4, IL-5, IL-10, IL-13, and IFN-γ after medium and OVA restimulation of lung cells. Graphs show results from 1 experiment with 5 mice per group (**Figure 2B**, PBS/PBS group in **Figures 2C–G**) or from 1 representative experiment from 2 independent experiments with 5 mice per group (groups PBS/OVA and TLA/OVA, **Figures 2C–G**). Error bars show mean ± SEM. *TLA*, tachyzoites lysate antigen; *OVA*, ovalbumin; *i.n.*, intranasal; *i.p.*, intraperitoneal; *macro*, macrophages; *eos*, eosinophils; *neutro*, neutrophils; *lympho*, lymphocytes; *nd*, not detectable; *ns*, not significant; *OD*, optical density; *P < 0.05; **P < 0.01; ***P < 0.001; ****P < 0.0001.

Mice were treated intranasally with TLA 30 min before each systemic application of OVA in alum on days 0 and 14 and before each intranasal challenge with OVA on days 21 to 23 (**Figure 2A**). Allergic airway hyperresponsiveness (**Figure 2B**) and eosinophilia (**Figure 2C**, **Supplementary Figure S1B**) were reduced in TLA-treated mice (TLA/OVA) compared to allergic controls (PBS/OVA). No effect on the levels of OVA-specific IgG2a in BALF was observed (**Figure 2D**). Although the TLA treatment did not reduce the histopathology score or the number of PAS-positive goblet cells in the lung (**Figures 2E, F** **)**, reduced levels of type 2 cytokines and IL-10 were measured in OVA-restimulated lung cell cultures of TLA/OVA mice in comparison to PBS/OVA controls (**Figure 2G**). Production of IFN-γ in the cultures was not affected by TLA-treatment (**Figure 2G**).

**Figure 3:**
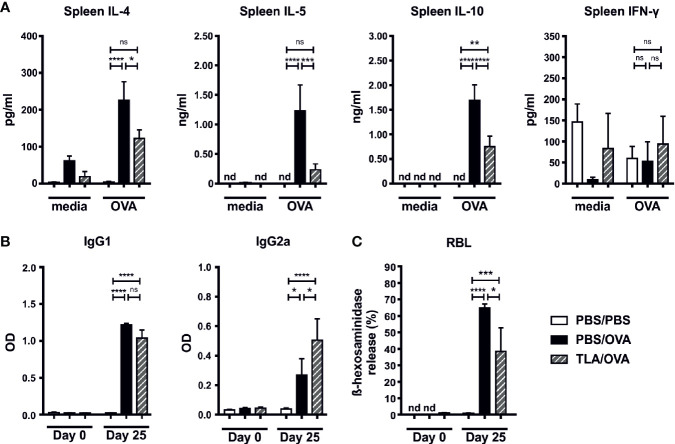
TLA reduces systemic IL-4 and serum IgE-levels in a co-application model. **(A)** Levels of IL-4, IL-5, IL-10, and IFN-γ after medium and ovalbumin (OVA) restimulation of spleen cells from mice treated as in **Figure 2A**. **(B)** Levels of OVA-specific antibodies IgG1 and IgG2a in serum collected at the beginning and at the end of the experiment. **(C)** Release of β-hexosaminidase by rat basophil leukemia (RBL) cells. Graphs show results from 1 experiment with 5 mice per group (PBS/PBS) or 1 representative experiment from 2 independent experiments with 5 mice per group (PBS/OVA and TLA/OVA). Error bars show mean ± SEM. *TLA*, tachyzoites lysate antigen; *OD*, optical density; *nd*, not detectable; *ns*, not significant; *P < 0.05; **P < 0.01; ***P < 0.001; ****P < 0.0001.

Similar to the local responses, OVA-restimulated spleen cells of TLA/OVA mice expressed lower levels of IL-4, IL-5, and IL-10 compared to PBS/OVA mice, while no significant difference was detected for IFN-γ (**Figure 3A**). In serum, OVA-specific levels of IgG1 were comparable between PBS/OVA and TLA/OVA mice, while OVA-specific IgG2a was increased upon TLA treatment compared to PBS/OVA mice (**Figure 3B**). Moreover, TLA led to a reduction of OVA-specific IgE-mediated release of β-hexosaminidase in RBL cells compared to PBS/OVA mice (**Figure 3C**).

### Therapeutic Intranasal TLA Application Reduces Th2 Responses in the Lung and Spleen

**Figure 4:**
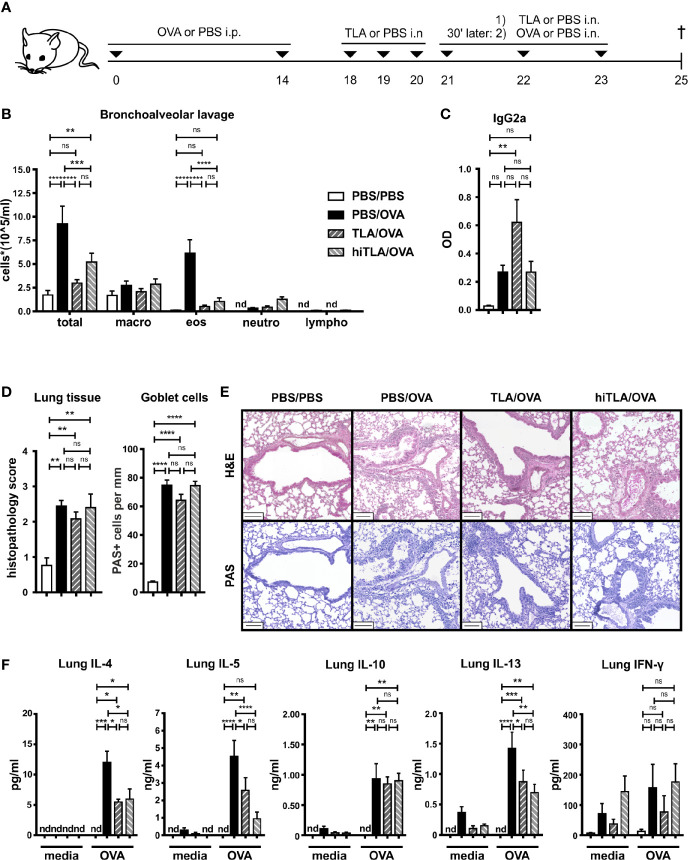
Therapeutic treatment with native or heat-inactivated TLA reduces allergic airway inflammation. **(A)** Experimental design. **(B)** Differential cell count in bronchoalveolar lavage (BALF). **(C)** Levels of OVA-specific antibody IgG2a in BALF collected at the end of the experiment. **(D)** Average histopathology score and number of Periodic acid-Schiff (PAS)-positive goblet cells of lung sections. **(E)** PAS or H&E-stained lung sections from 1 representative example from each group (n = 5); scale bars, 100 µm. **(F)** Levels of IL-4, IL-5, IL-10, IL-13, and IFN-γ after medium and OVA restimulation of lung cells. Graphs show results from 1 representative experiment from 2 independent experiments with 5 mice per group. Error bars show mean ± SEM. *TLA*, tachyzoites lysate antigen; *hiTLA*, heat-inactivated; OVA, ovalbumin; i.n., intranasal; i.p., intraperitoneal; macro, macrophages; eos, eosinophils; neutro, neutrophils; lympho, lymphocytes; n.s., not significant; OD, optical density; *P < 0.05; **P < 0.01; ***P < 0.001; ****P < 0.0001.

**Figure 5:**
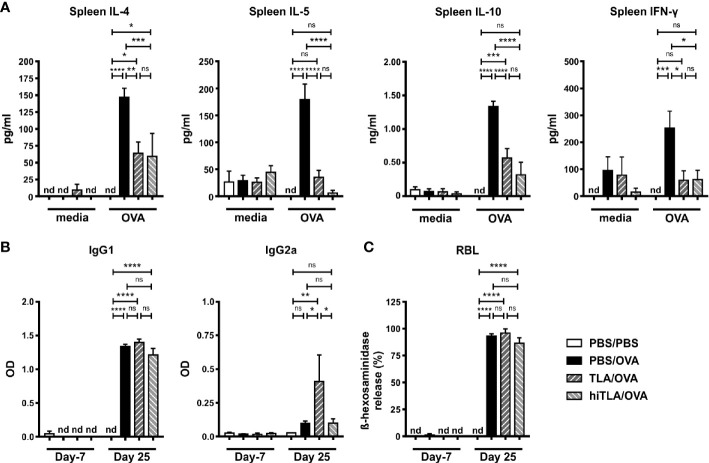
Therapeutic treatment with native or heat-inactivated TLA reduces systemic type 2 responses. **(A)** Levels of IL-4, IL-5, IL-10, and IFN-γ after medium and ovalbumin (OVA) restimulation of spleen cells from mice treated as in **Figure 4A**. **(B)** Levels of OVA-specific antibodies IgG1 and IgG2a in serum collected at the beginning and at the end of the experiment. **(C)** Release of β-hexosaminidase by rat basophil leukemia (RBL) cells. Graphs show results from 1 representative experiment from 2 independent experiments with 5 mice per group. Error bars show mean ± SEM. *nd*, not detectable; *TLA*, tachyzoites lysate antigen; *hiTLA*, heat-inactivated TLA; *ns*, not significant; *OD*, optical density; *P < 0.05; **P < 0.01; ***P < 0.001; ****P < 0.0001.

Next, we investigated whether also a therapeutic application of TLA reduces allergic responses. Additionally, we tested if heat-inactivation of TLA impairs its immunomodulatory effects. Sensitized mice were treated intranasally with native TLA on days 18 to 23 (**Figure 4A**). Another group of mice received heat-inactivated TLA (hiTLA/OVA). Both groups that were treated with TLA and hiTLA exhibited reduced numbers of eosinophils in the BALF (**Figure 4B** **,** **Supplementary Figure S1C**), but no changes were seen in the levels of OVA-specific IgG2a in BALF, in degree of histopathology scores or in the number of PAS-positive goblet cells in the lung (**Figures 4C–E**) in comparison to allergic controls. The production of type 2 cytokines was reduced in OVA-restimulated lung cells of TLA/OVA and hiTLA/OVA mice compared to PBS/OVA mice, while no effect was seen on the production of IL-10 and IFN-γ (**Figure 4F**). Similarly, levels of type 2 cytokines and also IL-10 were reduced in OVA-restimulated spleen cells of both TLA/OVA and hiTLA/OVA mice compared to PBS/OVA mice (**Figure 5A**). Levels of IFN-γ in OVA-restimulated spleen cells of TLA/OVA and hiTLA/OVA treated groups was comparable with sham treated mice and reduced compared to allergic controls (**Figure 5A**). Levels of OVA-specific IgG1 in serum were comparable between TLA/OVA, hiTLA/OVA and PBS/OVA mice (**Figure 5B**). TLA treatment increased OVA-specific IgG2a levels in serum compared to PBS/OVA and hiTLA/OVA mice (**Figure 5B**). There was no difference in OVA-specific IgE-mediated β-hexosaminidase release from RBL in serum samples between all allergen-exposed groups (**Figure 5C**).

### Characterization of TLA With Lectins Reveals a Complex Pattern of Epitope Sugars

**Figure 6:**
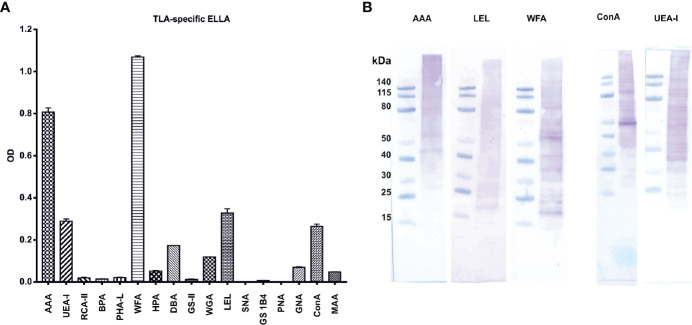
Characterization of TLA with enzyme-linked lectin assay (ELLA) and lectin-Western blots reveal a complex pattern of epitope sugars: **(A)** abundance of tachyzoite lysate antigen’s (TLA)s epitope sugars evaluated with ELLA. **(B)** Distribution of epitope sugars on proteins and peptides of TLA. *AAA Anguilla anguilla* agglutinin; *UEA-I Ulex europaeus* agglutinin-I; *RCA-II Ricinus communis* agglutinin II; *BPA Bauhinia purpurea* agglutinin; *PHA-L* phytohemagglutinin; *WFA Wisteria floribunda* agglutinin; *HPA Helix pomatia* agglutinin; *DBA Dolichos biflorus* agglutinin; *GS-II Griffonia simplicifolia* agglutinin; *WGA* Wheat germ agglutinin; *LEL Lycopersicon esculentum* lectin; *SNA Sambucus nigra* agglutinin; *GS1B4 Griffonia simplifolica*-1B4; *PNA* peanut agglutinin; *GNA Galanthus nivalis* agglutinin; *ConA* Concanavalin A; *MAA Maackia amurensis* agglutinin. *TLA*, tachyzoites lysate antigen; *OD*, optical density.

The immunomodulatory properties of native and hiTLA were comparable as shown in **Figures 4** and **5**, suggesting that not proteins, but rather heat-stable components, such as carbohydrates, might play a role in immunomodulation. Hence, we next characterized the diversity and abundance of epitope sugars with ELLA. Several lectins bound to TLA, indicating a complex glycosylation pattern. WFA, (specifically binding to β-GalNAc), followed by AAA, LEL, UEA-I, and ConA, (specifically binding to α-Fuc, β-GlcNAc, α-Fuc/Arabinose, and α-Man/α-Glc, respectively) showed the highest absorbance, suggesting that these epitope sugars are the most prevalent in TLA (**Figure 6A**). Based on the ELLA results, we performed Western blotting of TLA probed with lectins which exhibited the highest signal when measured by ELLA (**Figure 6B**). The lectin UEA-I was binding to proteins with a size between 40 and 115 kDA, while AAA was binding to proteins with a size of approximately more than 60 kDa. LEL-binding was distributed evenly between smaller and larger proteins and WFA-binding was detected on distinct bands of smaller and mid-sized proteins ranging from approximately 20 to 80 kDa. By probing with ConA, Western blot analysis showed a strong band at approximately 50 kDa. Additional proteins covered with α-Man/α-Glc epitope sugars were detected from 30 kDa size upwards.

### Native and Heat-Inactivated, But Not Deglycosylated TLA Reduced Type 2 Cytokines in Splenocytes of Allergic Mice *Ex Vivo*

**Figure 7:**
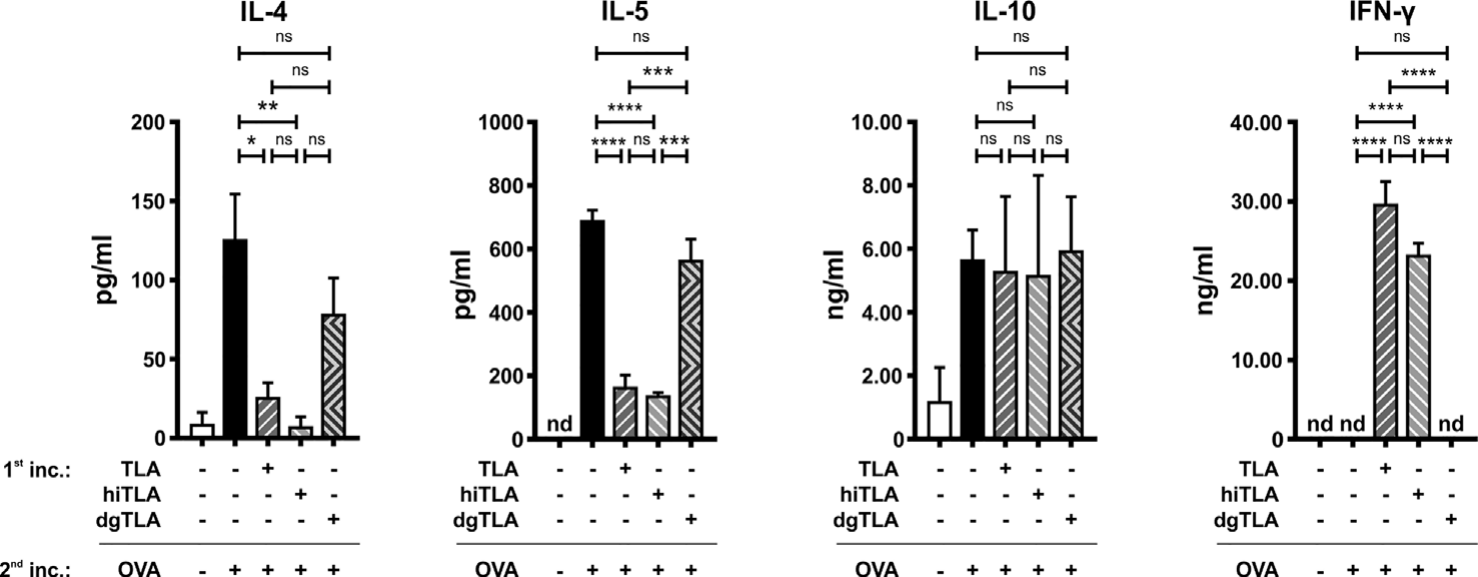
*Ex vivo* stimulation with TLA and hiTLA, but not dgTLA reduces type 2 cytokines and elevates IFN-γ. Levels of IL-4, IL-5, IL-10, and IFN-γ after stimulation with medium, TLA, hiTLA or dgTLA, followed by stimulation with medium or ovalbumin (OVA) of splenocytes excised from allergic control mice after sensitization and challenge. Graphs show results from 1 representative experiment from 2 independent experiments with 3 mice per group. Error bars show mean ± SEM. *nd* not detectable; *TLA*, tachyzoites lysate antigen; *hiTLA*, heat-inactivated TLA; *dgTLA*, deglycosylated TLA; *inc*, incubation. *ns*, not significant; *P < 0.05; **P < 0.01; ***P < 0.001; ****P < 0.0001.

Next, we tested whether deglycosylation of TLA impairs its immunomodulatory properties. The effects of TLA, hiTLA, and dgTLA on spleens of allergic mice were examined *ex vivo*. The removal of glycan moieties in dgTLA was verified with a ConA-specific Western blot (data not shown). Single-cell suspensions of allergic spleens were pre-incubated 60 min either with media, native TLA, hiTLA or dgTLA followed by 72 h restimulation with either media (only for non-stimulated cells) or OVA. Pre-incubation with TLA and hiTLA, but not with dgTLA, followed by restimulation with OVA reduced levels of OVA-specific IL-4 and IL-5 compared to sham-treated controls (**Figure 7**). While the levels of IL-10 were comparable between all groups, the production of IFN-γ was increased after pre-incubation with TLA and hiTLA, followed by restimulation of OVA compared to media/OVA, while pre-incubation with dgTLA had no effect on the levels of IFN-γ (**Figure 7**).

## Discussion

Epidemiological studies have shown inverse associations between allergy and infection with *T. gondii* (20, 42, 43). These findings were confirmed in experimental settings, where we and others have shown that infection with *T. gondii* (21, 22) or intraperitoneal injection of extracts of different *T*. *gondii* developmental stages, such as oocysts and tachyzoites, admixed to a potent adjuvant, such as alum or Freund’s complete adjuvant, prevented allergy in mice (23, 24). Here we show that the less invasive mucosal application of TLA *via* the nose reduced the development of allergic airway inflammation in mice. Importantly, the beneficial effect was achieved by co-application of TLA during the allergic sensitization and challenge as well as by therapeutic treatment in sensitized mice. Of note, TLA was applied in the absence of any adjuvant.

To induce tolerance by immunomodulatory substances, the mucosal route of application is advantageous over the parenteral route for several reasons: i) the active compound is absorbed slower when applied *via* the mucosa compared to parenteral application which might be beneficial for achievement of a sustained protective effect, ii) the needle-free application allows for easy self-administration, and iii) the mucosal route tolerates lower purity of the drug compared to the parenteral route (44, 45). The preferred mucosal site of the application, either the nose, mouth, or vagina, may vary depending on various factors, such as the frequency of administration, the desired drug dosage and formulation, or the target organ (46). In our present study, the intranasal TLA application reduced the development of allergy in the lung, but also modulated systemic immune responses, such as levels of allergen-specific antibodies in serum or allergen-specific recall responses in the spleen. These results are in agreement with previous findings, where intranasal application of recombinant probiotic *Escherichia coli* Nissle 1917 prevented allergic airway inflammation in mice accompanied by reduced local and systemic allergic responses (30). Similarly, nasal vaccines induced protective local and systemic responses in humans (47).

We and others have shown previously that mice which underwent allergic sensitization and challenge in the course of *T. gondii* infection were protected from the development of allergy (22, 48). Here we show that allergic sensitization and challenge in the close proximity to TLA-exposure had a similar protective effect. However, when mice were treated with TLA seven to five days before the beginning of the sensitization, the development of allergy was not prevented. Altogether, these observations suggest that in order to reach mucosally-induced immunological tolerance, the host needs to be exposed to the *T. gondii* immunomodulatory molecules during the allergen exposure, suggesting a possible application of TLA in seasonal treatment protocols.

Generally, it is more challenging to cure patients with established allergic diseases compared to prophylactic approaches (49). We show that intranasal application of TLA to sensitized mice exclusively during intranasal challenge with OVA reduced local as well as systemic allergic responses. The sensitization status was confirmed by increased levels of OVA-specific IgG1 in serum and elevated IgE-mediated β-hexosaminidase release by RBL cells in comparison to sham-treated controls (data not shown). In contrast to our previous study, where therapeutic intranasal treatment with *E. coli* O83 failed to reduce eosinophilia (29), therapeutic TLA application reduced the recruitment of eosinophils to the lungs. We hypothesize that the protective effect of TLA in adult mice lasts only for a limited time and repetitive applications might be required to reach a prolonged protective effect. However, the temporary character of suppressed type 2 responses might be favorable to treat patients suffering from seasonal allergies. For example, birch or grass pollen allergic patients are exposed to the relevant allergens only during the pollen season (50), thus, the therapeutic application of immunomodulators exclusively during this period could be sufficient to reduce the disease progression.

The significance of IFN-γ in resolving allergy was highlighted by Coyle et al. (51), showing that IFN-γ receptor knockout mice exhibited prolonged eosinophilia compared to wild type controls (51). IFN-γ is also a crucial player in host defense to control an infection with *T. gondii* (52, 53) and extracts derived from this parasite, such as TLA or OLA, are potent inducers of this cytokine both *in vivo* and *in vitro* (23, 24). In a mouse model of allergy, we have previously demonstrated that infection with *T. gondii* or intraperitoneal application of TLA admixed to OVA in alum reduced type 2 responses accompanied by increased levels of allergen-specific IFN-γ in restimulated splenocytes compared to allergic controls (22, 24). Similarly, Fenoy et al. (48) detected increased levels of OVA-specific IFN-γ in sensitized and challenged mice during acute *T. gondii* infection, but the production of IFN-γ was decreased in chronically infected mice.

Of note, mucosal application of *Lactobacillus paracasei* NCC 2461, a probiotic bacterial strain which is a potent inducer of IFN-γ *in vitro*, reduced not only allergic type 2 responses, but also allergen-specific IFN-γ in restimulated splenocytes (31, 54). Here we show that TLA increased levels of OVA-specific type 1 isotype IgG2a in serum in the co-application and the treatment protocol. As the BALF closely reflects the immunological processes in the lung, we also measured levels of OVA-specific IgG2a and levels of IFN-γ and IL-17AF in BALF. The data show that TLA did not influence the local type 1 responses (data for IFN-γ and IL-17AF not shown).

The success of SIT in human patients is *inter alia* reflected by a reduction of allergen-specific IgE (55, 56). Here, we show that TLA, applied concurrently with sensitization and challenge, reduced the activity of OVA-specific IgE in serum in comparison to controls. However, the reduction of IgE-dependent β-hexosaminidase release by RBL cells was not observed in mice, where i) the interval between the last TLA-treatment and the start of sensitization was five days or ii) the treatment was applied to mice with completed sensitization. Although the therapeutic TLA application did not reduce the β-hexosaminidase release, the treatment reduced the recruitment of eosinophils to the lung and production of allergen-specific type 2 cytokines in the lung and spleen. Similarly, it has been shown that, although SIT led to clinical improvement in allergic patients, the levels of IgE remained initially high and declined gradually over months or even years (56). Here, in the therapeutic experiment, serum was collected already seven days after the TLA was first introduced to mice, and thus the question remains whether analysis of sera collected at a later time point or after prolonged treatment with TLA would lead to a reduction of humoral type 2 responses in sensitized animals.

It has been shown that carbohydrates and carbohydrate-binding proteins play an essential role in adhesion or invasion of certain parasites into host cells (57). Furthermore, parasite-derived carbohydrates can trigger the host´s innate and adaptive immune responses. *Toxoplasma* assembles polysaccharides and many cellular proteins and lipids are glycosylated. A recent study revealed that the *Toxoplasma* genome encodes a set of predicted glycogenes with a possible role in assembling of N-glycans, O-glycans, a C-glycan, GPI-anchors, and polysaccharides (58). Applying a double-CRISPR/Cas9 strategy indicated an important role of certain glycan-biosynthesizing enzymes (e.g. glycosyltransferase responsible for assembling novel Glc-Fuc–type O-glycans) for *in vitro* growth of *Toxoplasma* (58).

Here, we applied ELLA, a lectin ELISA, to characterize the glycosylation pattern of TLA. ELLA is commonly used for analysis of glycoconjugates and was previously employed to determine immunogenic epitope sugars of parasites, such as *Tritrichomonas foetus* or *Trypanosoma cruzi* (59, 60). Our data showed a strong binding of the lectins WFA, AAA, ConA, and LEL to TLA, which indicate high abundances of terminal GalNAc, Fuc, Man/Glc, and GlcNAc, respectively. Previously, terminal Fuc(1→4)GlcNAc as well as the terminal motives Fuc(1→3)GalNAc and Fuc1→4(Fuc1→3)GlcNAc, which are commonly found in *S. mansoni eggs*, were shown to be immunogenic (61, 62). Furthermore, terminal fucosylation was shown to play a critical role in colonization and initial evasion of the host’s immune system in infections with *Helicobacter pylori* (63). On the other hand, it was suggested that mannose plays a pivotal role in the virulence of *Leishmania mexicana* (64). Host cells, such as dendritic cells, Langerhans cells, and lymphocytes, express a plethora of carbohydrate-binding proteins including C-type lectins, selectins, and galectins which may recognize and interact with parasite-derived glycosylation patterns (65, 66).

We have shown previously *in vitro* that heat–inactivation of TLA does not affect its immunomodulatory properties (24). Here, we confirmed this observation *in vivo* and additionally, by using an *ex vivo* model, we could show that deglycosylation of TLA abolished its anti-allergic effects. However, more studies are needed to understand the precise role of *T. gondii* carbohydrates in allergy prevention.

In addition to carbohydrates, also *T. gondii*-specific lipids play an essential role for interacting with host cells and the host’s immune system resulting in the establishment and maintenance of long-term persistence in the host (67). For example, fatty acids present in *T. gondii* tachyzoites exhibit immunomodulatory effects on murine macrophages (68). However, the link between the *T. gondii* lipidome and allergy prevention remains to be elucidated.

Although clinical trials using infections with nematode parasites opened new possibilities to treat immune-mediated inflammatory diseases in humans (69), the ultimate goal is the use of parasite-derived extracts or single molecules to replicate the immunomodulatory effect of the infection without causing any of the obvious disadvantages. Indeed, promising results have been seen in studies where parasite products were administered in mouse models (24, 70, 71), but to date no parasite-derived molecules have been applied as a treatment for humans yet. Molecules present in TLA have evolved to act in the environment of the host´s immune system and thus represent natural biologicals with high potential in future translational research.

## Conclusion

In the present study, we show that mucosal application of TLA during sensitization and challenge, as well as therapeutic treatment of sensitized mice, reduces allergic exacerbations in a mouse model of allergic airway inflammation. Additionally, we show that TLA is highly glycosylated and that removal of carbohydrates impaired its immunomodulatory properties. We therefore believe that further investigations focusing on glycosylation patterns of TLA will strengthen our understanding of host-parasite interactions and will pave the way for novel, therapeutic treatment strategies against allergic disorders.

## Data Availability Statement

The datasets presented in this article are not readily available because the raw data supporting the conclusions of this article will be made available by the authors upon reasonable request. Requests to access the datasets should be directed to IS, irma.schabussova@meduniwien.ac.at.

## Ethics Statement

The animal study was reviewed and approved by the Ethics Committee of the Medical University of Vienna and the Austrian Federal Ministry of Education, Science and Research (BMWFW-66.009/0358-WF/V/3b/2015 and BMBWF-66.009/0277-V/3b/2019).

## Author Contributions

IS and UW designed and supervised the study and acquired funding. EK and IS prepared documents for ethics approval. RP cultivated *T. gondii* tachyzoites. MD and EK prepared TLA. EK performed experiments, analyzed and interpreted the data. NG performed histological analysis of lung tissue samples. EK and IS wrote the initial draft of the manuscript. EK provided visualization of the data. EK, IS, AI-K, AJ, MD, and AW critically reviewed and edited the manuscript. IS, UW, and AJ provided resources. All authors contributed to the article and approved the submitted version.

## Funding

This work was supported by the Austrian Science Fund (FWF) grants SFB F4612 and the OeAD grants CZ 17/2019, CZ 16/2019, SRB 20/2018, and PL 04/2019 (to EK and IS).

## Conflict of Interest

The authors declare that the research was conducted in the absence of any commercial or financial relationships that could be construed as a potential conflict of interest.
