# Supplementary material for: Reduction of Allergic Lung Disease by Mucosal Application of Toxoplasma gondii-Derived Molecules: Possible Role of Carbohydrates
Source: Front Immunol. 2021 Mar 10;11:612766. doi: 10.3389/fimmu.2020.612766 (PMC7988086; doi:10.3389/fimmu.2020.612766)
Supplement: Supplementary file 1 [file DataSheet_1.pdf]

Supplementary Material

1. Supplementary Figures

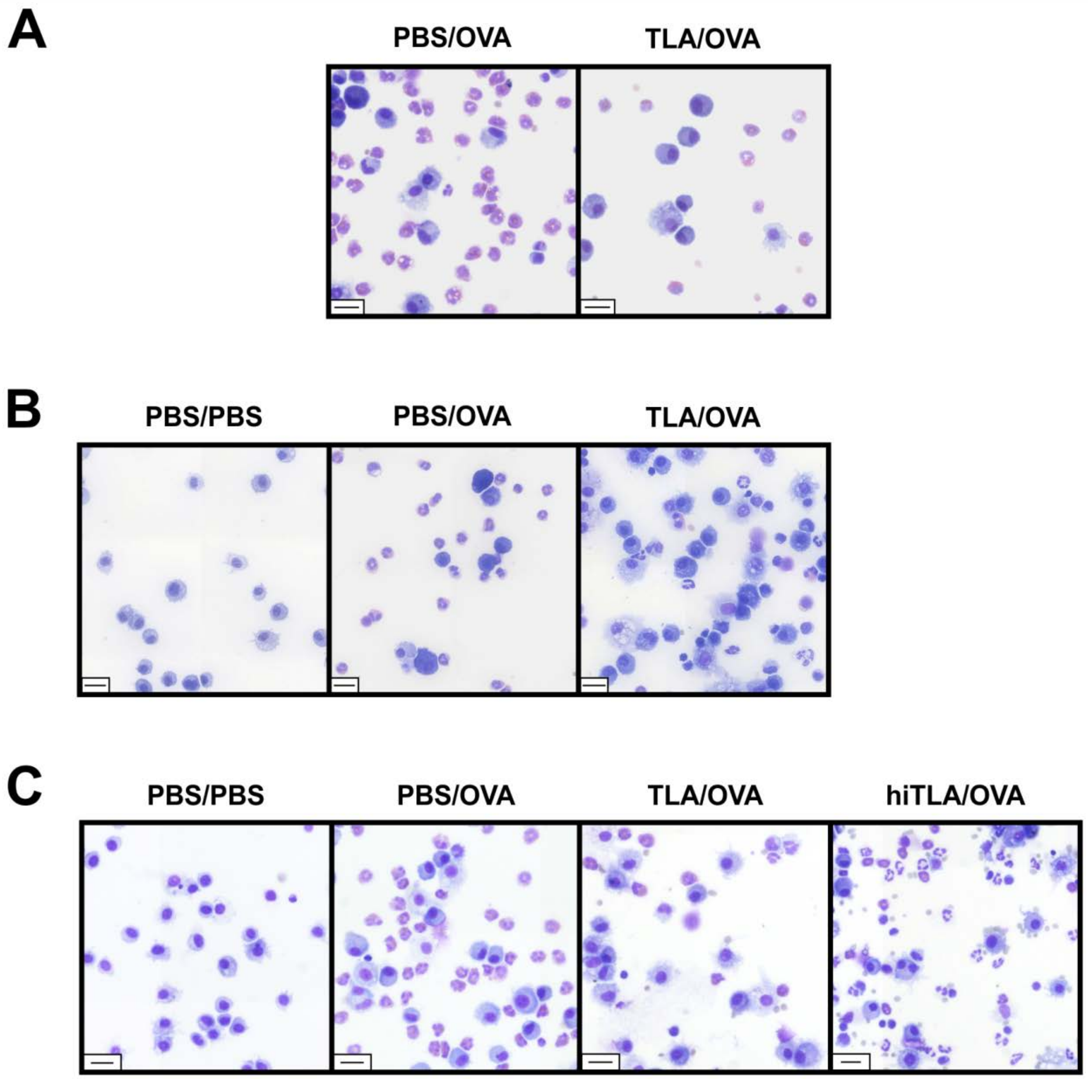

Supplementary Figure S1. H&E-stained cytospins from BALF. Images show 1 representative example from each group (n = 5) from the prophylactic model **(A)**, the co-application model **(B)**, and the therapeutic model **(C)**. Scale bar shows 20 µm.
